# Gla-Rich Protein (GRP) as an Early and Novel Marker of Vascular Calcification and Kidney Dysfunction in Diabetic Patients with CKD: A Pilot Cross-Sectional Study

**DOI:** 10.3390/jcm9030635

**Published:** 2020-02-27

**Authors:** Ana P. Silva, Carla S.B. Viegas, Filipa Mendes, Ana Macedo, Patrícia Guilherme, Nelson Tavares, Carolina Dias, Fátima Rato, Nélio Santos, Marília Faísca, Edgar de Almeida, Pedro L. Neves, Dina C. Simes

**Affiliations:** 1 Department of Nephrology, Centro Hospitalar Universitário do Algarve, 8000-386 Faro, Portugal; anapassionara@gmail.com (A.P.S.); filipabritomendes@gmail.com (F.M.); pleaon@hotmail.com (P.L.N.); 2 Department of Biomedical Sciences and Medicine, Universidade do Algarve, 8005-139 Faro, Portugal; amacedo@keypoint.pt (A.M.); Heycarol.5@gmail.com (C.D.); 3 Centre of Marine Sciences (CCMAR), Universidade do Algarve, 8005-139 Faro, Portugal; caviegas@ualg.pt; 4 GenoGla Diagnostics, Centre of Marine Sciences (CCMAR), Universidade do Algarve, 8005-139 Faro, Portugal; 5 Keypoint Group, 1495-190 Miraflores, Portugal; 6 Department of Cardiology, Centro Hospitalar Universitário do Algarve, 8000-386 Faro, Portugal; cpguilherme@gmail.com (P.G.); nelson.tavares63@gmail.com (N.T.); 7 Pathology Clinic, Centro Hospitalar Universitário do Algarve, 8000-386 Faro, Portugal; fatima.rato@gmail.com (F.R.); neliofilipe.santos@gmail.com (N.S.); marilia.faisca@synlab.pt (M.F.); 8 Faculdade de Medicina da Universidade de Lisboa, 1600-190 Lisboa, Portugal; edealmeida@mail.telepac.pt

**Keywords:** chronic kidney disease, cardiovascular disease, cardiovascular calcification, cardiovascular risk assessment, Gla-rich protein, vascular calcification inhibitors

## Abstract

Vascular calcification (VC) is one of the strongest predictors of cardiovascular risk in chronic kidney disease (CKD) patients. New diagnostic/prognostic tools are required for early detection of VC allowing interventional strategies. Gla-rich protein (GRP) is a cardiovascular calcification inhibitor, whose clinical utility is here highlighted. The present study explores, for the first time, correlations between levels of GRP in serum with CKD developmental stage, mineral metabolism markers, VC and pulse pressure (PP), in a cohort of 80 diabetic patients with mild to moderate CKD (stages 2–4). Spearman’s correlation analysis revealed a positive association of GRP serum levels with estimated glomerular filtration rate (eGFR) and α-Klotho, while a negative correlation with phosphate (P), fibroblast growth factor 23 (FGF-23), vascular calcification score (VCS), PP, calcium (x) phosphate (CaxP) and interleukin 6 (IL-6). Serum GRP levels were found to progressively decrease from stage 2 to stage 4 CKD. Multivariate analysis identified low levels of eGFR and GRP, and high levels of FGF-23 associated with both the VCS and PP. These results indicate an association between GRP, renal dysfunction and CKD-mineral and bone disorder. The relationship between low levels of GRP and vascular calcifications suggests a future, potential utility for GRP as an early marker of vascular damage in CKD.

## 1. Introduction

Chronic kidney disease (CKD) is estimated to affect more than 10% of the global population and represents an increasing health and economic burden for the society [1,2]. Cardiovascular disease (CVD) is the most important complication of CKD and the primary cause of death in these patients [3]. In addition to traditional risk factors, most patients with CKD display abnormal mineral metabolism (MM) with underlying hormonal dysregulation, defined as chronic kidney disease-mineral and bone disorder (CKD-MBD) [4]. CKD-MBD involves changes in mineral ion homeostasis, bone quality and turnover, cardiovascular and soft tissue calcifications, which highly contribute for cardiovascular complications [4,5]. Vascular calcification (VC) is associated with significant morbidity and mortality and a strong predictor of cardiovascular risk in CKD patients [6,7]. The prevalence of VC and the risk of CVD are shown to increase as glomerular filtration rate (GFR) declines in CKD patients [3,8]. In fact, bone MM abnormalities start during the first stages of CKD, long before renal replacement therapy is required [9]. Cardiovascular calcification is a highly-controlled and regulated process of calcium phosphate mineral deposition in the intima and media layers of the vessel wall and in cardiac valves. Epidemiologically, CKD, diabetes mellitus and atherosclerosis are the clinical conditions that most contribute towards development of vascular and valves calcification [10]. Increased vascular stiffness is an established independent predictor of cardiovascular morbidity and mortality [11,12], and aortic calcification has been positively associated with arterial stiffness in the healthy and CKD populations [13,14]. Increased pulse pressure (PP) is one of the most evident hemodynamic consequences of increased vascular stiffness, and has been suggested as correlated with arterial calcification and cardiovascular events in non-CKD, dialysis and non-dialysis patients [15,16]. Although the relevance of vascular calcification assessment is recognized in clinical practice, most reliable quantitative methods are still radiographic or echographic related, with many shortcomings, such as cost and time consumption, particular in the case of computed tomography methods, radiation exposure, operator dependency and lack of standardized scores [17]. Therefore, the development of biomarkers for early detection of VC are crucial for the prevention of CVD outcomes in CKD patients, allowing preventive measures to reduce the development and progression of VC, left ventricular hypertrophy and arterial stiffness.

VC is a highly-controlled multifactorial process that requires constant inhibition [18]. High prevalence of VC in CKD patients is suggested to result from several interconnected processes involving dysregulation of endogenous calcification inhibitors, abnormal mineral metabolism and inflammation [19]. Biochemical targets known to be involved in these pathological processes have been explored for their potential use as biomarkers for VC and cardiovascular risk assessment [19].

Gla-rich protein (GRP), also known as upper zone of growth plate and cartilage matrix associated protein (UCMA) [20], is a circulating vitamin K-dependent protein (VKDP) with a dual capacity to function as an inhibitor of pathological calcification and anti-inflammatory agent, in the articular and cardiovascular systems [21,22,23,24]. Although GRP has been suggested as a potential marker for VC, its clinical utility has never been shown. Here we explored, for the first time, the relationship between levels of circulating GRP and chronic renal dysfunction, mineral metabolism and vascular calcifications assessed by the simple vascular calcification score (VCS) and pulse pressure (PP) in a cohort of diabetic patients with mild to moderate CKD (stages 2–4).

## 2. Experimental Section

### 2.1. Patient Selection

This cross-sectional study was conducted in the outpatient diabetic nephropathy clinic of the Centro Hospitalar Universitário do Algarve in Faro, Portugal, from 2012 to 2017, enrolling 107 consecutive adult type 2 diabetic Caucasian patients with stages 2–4 CKD; 27 participants did not meet the inclusion criteria and were excluded. A total of 80 participants were involved. The study was approved by the ethics committee of the hospital; all principles of the Declaration of Helsinki were followed; and written informed consent was obtained from all patients. Diabetes classification was based on the guidelines from the American Diabetes Association [25]. The exclusion criteria were: Age > 65 years; previous CVD as described [26]; changes in the GFR >30% (last 3 months); changes in antihypertensive therapy (last 2 weeks), uncontrolled hypertension (BP ≥ 140/90 mmHg); albumin/creatinine ratio (ACR) ≥ 500 mg/g (assessed twice in 3 months); eGFR ≤ 15 mL/min/1.73 m^2^ or ≥ 90 mL/min/1.73 m^2^; parathyroid hormone (PTH) ≥ 350 pg/mL; phosphate (P) > 5.5 mg/dL; patients on anticoagulant therapies; type 1 diabetes; non-diabetic renal disease; neoplastic or infectious diseases. Demographic, clinical, laboratory results and medication data were collected from the clinical records.

### 2.2. Laboratory Measurements

Fasting blood samples were drawn from all subjects and plasma/serum was frozen at -80 ºC in order to measure eGFR, P, calcium (Ca), PTH, glycated hemoglobin (HbA1c), interleukin 6 (IL-6), fibroblast growth factor 23 (FGF-23) and soluble α-Klotho, as described [26,27]. Serum levels of GRP were determined using a recently developed sandwich ELISA assay for the quantification of total GRP protein forms [24]. Blinded measurements of GRP levels were performed at GenoGla Diagnostics, University of Algarve, Faro, Portugal. ACR was determined as described [26].

### 2.3. Pulse Pressure

Blood pressure (BP) was determined with oscillometric methods, with the patient in dorsal decubitus. Three measurements were taken with an interval of 5 min. Pulse pressure (PP) was calculated as the difference between the systolic blood pressure and the diastolic blood pressure. Increased cardiovascular risk was considered for PP values greater than 50 mmHg.

### 2.4. Cardiovascular Calcification Measurements

The assessment of simple vascular calcifications was performed using the plain X-ray of the hands and pelvis (Adragão score) and nominated as the vascular calcification score (VCS). Increased cardiovascular risk was considered for VCS ≥ 3 [28].

### 2.5. Statistical Analysis

Descriptive results were presented using mean and standard deviation (± SD) for continuous variables with normal distribution, using the Kolmogorov–Smirnov test. Categorical variables were described using absolute and relative frequencies. Categorical variables were compared using chi-squared test. Correlations between GRP and renal function, osteo-mineral markers, inflammation and vascular calcification parameters (VCS and PP) were evaluated by applying Spearman’s correlation test. Partial correlations were used to analyze relationships between GRP with renal function, vascular calcifications (VCS) and pulse pressure (PP), adjusted by sex and age groups. The association of GRP with eGFR was also evaluated by using simple linear regression analysis. For comparison between the stages of renal disease and serum GRP levels, ANOVA and a post hoc analysis with Scheffe test was used. CKD stages were defined by the eGFR (mL/min/1.73m^2^) for stage 2 (60–89), stage 3 (30–59) and for stage 4 (29–15) [29]. To assess the influence of tested parameters in GRP levels, the forward stepwise multiple linear regression analysis was used, with the covariates age, gender, eGFR, FGF-23, IL-6, P, CaxP, α-Klotho, VCS and PP.

Univariate logistic regression analysis was used to identify independent factors associated with the VCS and PP. Statistically significant variables were analyzed in multivariate logistic regression models (with forward stepwise selection with likelihood ratio) to assess the main predictive risk factors for VCS and PP. The variables were removed from the model when the *p*-value exceeded 0.10. Factors that remained significant at the 0.05 level in the multivariable models were considered to be significant contributors and were kept in the model. Potential confounding factors offered to the logistic regression models included age, gender, eGFR, FGF-23, IL-6, P, CaxP, α-Klotho and GRP. The exponentials of the model parameters were the adjusted odds ratio (ORa) to other variables of the model, with 95% confidence interval. The null hypothesis was rejected below the level of 5%. Statistical software SPSS (IBM Corp, Armonk, NY, USA; version 17.0), and GraphPad Prism version 8.0.0 (GraphPad Software, San Diego, California, USA) for Windows was used for statistical data analysis and graph design.

## 3. Results

The study enrolled 80 consenting patients meeting the inclusion criteria with stages 2–4 CKD, 28.7 % females, mean age of 56 ± 8.1 years (range: 41–65). All variables had a normal distribution. The mean GRP levels was 0.9 ± 0.56 ng/mL (range, 0.19–2.6 ng/mL). Table 1 describes the patients main clinical and biochemical characteristics, including osteo-mineral markers and vascular calcification parameters. A total of 47.5 % of patients presented a VCS ≥ 3 (28 males and 10 females) and 28.7 % had PP ≥ 50 mmHg.

To evaluate the association of GRP with renal function, osteo-mineral markers and vascular calcification parameters, Spearman’s correlation analysis was performed using the variables age, eGFR, P, Ca, CaxP, PTH, FGF-23, α-Klotho, IL-6, PP and the VCS. The results revealed statistically significant strong positive correlations between GRP serum levels and eGFR (r = 0.863, *p* < 0.0001) and α-Klotho (r = 0.647, *p* < 0.0001), strong negative correlations with P (r = −0.715, *p* < 0.0001), FGF23 (r = −0.676, *p* < 0.0001), VCS (r = −0.822, *p* < 0.0001) and PP (r = −0.533, *p* < 0.0001), and moderate negative correlations with CaxP (*r* = –0.302, *p* = 0.006) and IL-6 (*r* = –0.349, *p* = 0.002) (Table 2).

**Figure 1:**
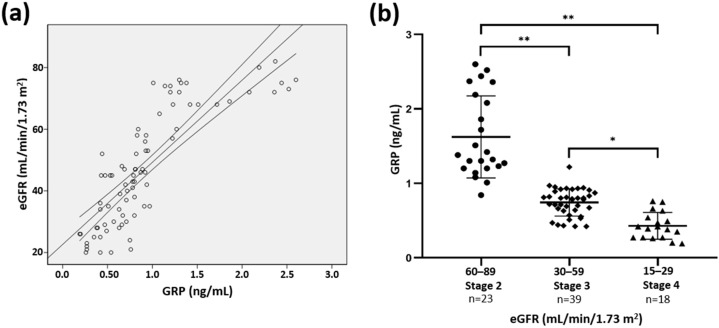
Association between serum Gla-rich protein (GRP) levels and kidney function. (**a**) The simple linear regression was used to assess the relationship between estimated glomerular filtration rate (eGFR) and serum GRP levels (β = 0.821; *p* < 0.0001). (**b**) Serum GRP levels divided by chronic kidney disease (CKD) stage. ANOVA test and a post hoc analysis with Scheffe test was used to analyse differences among the 3 groups (* *p* = 0.001, ** *p* < 0.0001).

A positive association between GRP serum levels and eGFR (β = 0.821; *p* < 0.0001) was also demonstrated (Figure 1a). Furthermore, GRP levels were shown to significantly decrease with deterioration of renal function from CKD stage 2 onward (Figure 1b). A correlation between GRP levels and eGFR remained significant after adjustments for age and gender (*r* = 0.823, *p* < 0.0001).

A forward stepwise multiple linear regression analysis, including all variables significantly correlated with GRP levels (Table 2), revealed that eGFR (β = 0.666; *p* < 0.0001) and the VCS (β = –0.238; *p* = 0.005) are the only factors influencing GRP levels.

Partial correlations between GRP levels, VCS and PP were analyzed after adjustments for age and gender. A strong negative correlation was found between GRP and the VCS (r = −0.677, *p* < 0.0001), and a moderate negative correlation with PP (r = −0.399, *p* < 0.0001), while a strong positive correlation was found between VCS and PP (r = 0.647, *p* < 0.0001) (Table 3).

Variables associated with the VCS and PP in univariate logistic regression analysis (Table 4) were used in multivariate logistic regression models. As shown in Table 5, only eGFR, GRP and FGF-23 were found significantly associated with both the VCS and PP.

## 4. Discussion

This is the first clinical study showing the association of circulating levels of GRP with CKD pathology and vascular calcification. Here we show that, in adult diabetic patients, serum GRP levels progressively decrease from stage 2 to stage 4 CKD, correlating with markers of mineral metabolism, vascular calcification and pulse pressure. Moreover, low levels of GRP were strongly associated with vascular calcification and pulse pressure, providing support to the hypothesis of being considered as a novel cardiovascular risk factor in this population.

Recently, a small study evaluating biomarkers of VC in hemodialysis patients after switch from traditional to online hemodiafiltration, included the measurement of serum GRP levels in their analysis [30]. In this prospective study no significant changes over time were observed for any of the VC biomarkers, including GRP. This emphasizes the current lack of representative clinical studies including information on circulating levels of GRP in human, and the importance of using validated GRP assays. The sandwich ELISA that was used in our study is based on a dual antibody system able to detect total GRP protein forms [24]. The antibodies included in this ELISA were previously validated and shown to be specific for GRP. The capture antibody was mapped to specifically recognize the N-terminal of human GRP-F1 isoform [24], while the detecting antibody has been validated for the specific detection of human GRP by immunohistochemical staining and Western blot, as described in previous studies [21,22,23,24]. Moreover, the suitability of this ELISA was previously validated and used to quantify GRP protein levels associated to circulating calciprotein particles (CPP) and extracellular vesicles (EVs) in serum samples of healthy and CKD individuals [24]. Although GRP is a γ-carboxylated protein, and other extra-hepatic VKDPs such as MGP and OC have been suggested of potential clinical use based on their γ-carboxylation status [31,32,33,34,35], our results clearly indicate that levels of total GRP in serum can be clinically relevant in a CKD context.

In our cohort study, a reduction in GRP levels associate with an increase in levels of the VC promoters P, FGF-23 and CaxP, and a decrease in the VC inhibitor α-Klotho, clearly showing a correlation between GRP and the dysregulation of phosphate metabolism characteristic of CKD-MBD. The relationship between bone mineral disorders and VC is well established and a major concern on the management of cardiovascular risk in the CKD population. In this complex interplay, the contribution of phosphate metabolism for VC and cardiovascular outcomes in CKD settings has been widely demonstrated [19,36]. Increased levels of serum P, FGF-23 and CaxP, and lower levels of α-Klotho, have all been associated with cardiovascular outcomes in the CKD population [19,36,37]. The association between high serum P levels and CKD is based on the role of phosphate as a primary stimulus for the osteochondrogenic transformation of VSMCs with calcifying capacity [36,38]. The association of GRP with CKD-MBD and VC are consistent with reported data regarding GRP functionality. GRP has been shown to be involved in VC inhibition at multiple levels, through the inhibition of VSMCs osteochondrogenic differentiation or the direct inhibition of mineral formation, maturation and growth, both in circulation and at the vascular tissue [21,24,39]. Complementary approaches of GRP add-of-function using a human *ex vivo* model of VC [21], and GRP depletion in VSMCs from GRP-/- mice [39], demonstrated the role of GRP as an inhibitor of extracellular matrix calcification and VSMCs osteochondrogenic differentiation. Although additional knowledge is required to fully elucidate the molecular mechanism(s) of GRP action in VC, it was demonstrated that GRP inhibit VSMCs osteochondrogenic differentiation with down-regulation of osteogenic markers, through direct binding to bone morphogenetic protein 2 (BMP2) [39]. Additionally, the mineralization competence of VSMCs-derived EVs, known as one of the major mechanisms of VC initiation at tissue level, was associated with decreased levels of GRP, indicating the importance of GRP in the early phases of VC [21]. Additionally, GRP was recently found as a constitutive component of circulating CPPs and EVs [24]. Decreased levels of GRP in CPPs and EVs from CKD stage 5 patients were associated with increased mineral maturation and increased potential to induce VSMCs calcification, by promoting cell osteochondrogenic differentiation and inflammation [24]. GRP was suggested as a link between systemic pro-calcific uremic conditions, including MM dysregulation, and VC at tissue level. Importantly, the protective role of GRP was clearly demonstrated when the calcification/osteogenic differentiation and inflammatory status induced in VSMCs were rescued by supplementation of CPPs isolated from CKD patients, with GRP [24].

While the involvement of GRP in multiple calcification-driving events clearly establish a biological rationale for the finding of a strong association between GRP levels and vascular calcifications, eGFR and VC were the only independent determinants of GRP levels in our population. In fact, a very strong correlation is shown between GRP and eGFR. Decrease in eGFR is accompanied by a decrease in GRP levels from CKD stage 2 to stage 4, indicating GRP as a possible early marker associated with renal dysfunction. Although the current study does not allow us to infer on the causality of this relationship, it has been demonstrated that the prevalence of VC increases as eGFR declines while low eGFR is associated with cardiovascular morbidity and mortality [40,41,42], and VC is a suggested link between low eGFR and worse cardiovascular outcome [8,9]. In concordance, our study shows that eGFR is associated with VC in our population. It is presently unclear whether the relationship between GRP and eGFR might be beyond VC, eventually involving kidney disease physiopathology. In addition, it is presently unknown whether reduced levels of GRP in serum have a causality relation with VC. Although increased GRP gene expression has been associated with increased calcification, increased protein accumulation has been detected at calcification sites, probably limiting its release into circulation [21]. Whether levels of GRP in circulation contribute to VC at the tissue level also requires further clarification. Low levels of GRP in circulating CPPs and EVs were shown to contribute to the modulation of vascular homeostasis, including calcification and inflammation [24]. Additional studies are required to understand this complex loop of interactions and clearly establish the molecular pathways leading to reduced levels of GRP in circulation, in situations of increased vascular calcification and decreased kidney function.

Limitations of our study include the small sample size, the fact that serum GRP levels were measured at a single point, and the absence of reference intervals for GRP levels in a healthy population. Despite the fact that levels of GRP in the general population are currently unknown, our present results showing decreased levels of serum GRP with decreased kidney function are in concordance with previous findings of decreased levels of GRP in circulating CPPs and EVs from CKD stage 5 patients relative to healthy controls [24]. This combined data supports the notion of an overall deficiency in circulating calcification inhibitors associated with CKD. This study included subjects with mild to moderate CKD followed in outpatient diabetic nephropathy clinic, and may not be representative of kidney disease of other etiology. The main strength of this study clearly resides in the novelty concerning the clinical utility of GRP. The presented data show that decreased levels of GRP in circulation parallels the progression of CKD and increased vascular calcification, suggesting a future potential use of GRP as an early marker of vascular damage in CKD patients.

## 5. Patents

The tools and methods described in this manuscript are included in a PCT patent application PCT/PT2009000046.

## Figures and Tables

**Table 1:** Baseline patient characteristics (n = 80).

General Characteristics	Values
Number of patients, *n*	80
Age (years)	56.0 ± 8.14
Gender (f/m)	24/56
BMI (Kg/m2)	23.4
Hb (g/dL)	12.97 ± 1.83
Albumin (g/dL)	4.27 ± 0.48
ACR (µg/mg)	137.37 ± 41.11
eGFR (mL/min per 1.73 m^2^)	47.26 ± 18.42
Phosphate (P) (mg/dL)	3.9 ± 0.67
Calcium (Ca) (mg/dL)	9.48 ± 0.62
Calcium (x) Phosphate (CaxP)	35.9 ± 5.8
PTH (pg/mL)	113.11 ± 74.65
FGF-23 (RU/mL)	135.32 ± 102.20
α-Klotho (pg/mL)	272.38.10 ± 169.95
IL-6 (pg/mL)	4.61 ± 2.60
GRP (ng/mL)	0.90 ± 0.56
HbA1c (%)	7.67 ± 1.47
Systolic BP (mmHg)	127.42 ± 8.56
Diastolic BP (mmHg)	78.58 ± 9.98
PP (mmHg)	45.65 ± 12.03
VCS (Adragão score)	2.7 ± 2.3
Diabetes-related CKD evolution time (months)	73.8 ± 8.7

BMI, body mass index; Hb, hemoglobin; ACR, urine albumin to creatinine ratio; eGFR, estimated glomerular filtration rate; PTH, parathyroid hormone; FGF-23, fibroblast growth factor 23; IL-6, interleukin 6; GRP, Gla-rich protein; HbA1c, glycated hemoglobin; BP, blood pressure; PP, pulse pressure; VCS, vascular calcification score.

**Table 2:** Correlation of GRP with renal function, osteo-mineral markers and vascular calcification.

Variables	r	*p* Value
Age	0.068	0.548
eGFR	0.863 **	<0.0001
P	–0.715 **	<0.0001
Ca	–0.124	0.273
CaxP	–0.302 **	0.006
PTH	0.113	0.317
FGF-23	–0.676 **	<0.0001
α-Klotho	0.647 **	<0.0001
IL-6	–0.349 **	0.002
VCS	–0.822 **	<0.0001
PP	–0.533 **	<0.0001

Spearman correlation coefficient (r). ** Correlation is significant at the 0.01 level (two-tailed). eGFR, estimated glomerular filtration rate; P, phosphate; Ca, calcium; CaxP, calcium (x) phosphate; PTH, parathyroid hormone; FGF-23, fibroblast growth factor 23; IL-6, interleukin 6; VCS, vascular calcification score; PP, pulse pressure.

**Table 3:** Partial correlation analysis between GRP, vascular calcification score (VCS) and pulse pressure (PP) after adjustments for age and gender.

Variables	GRP		VCS		PP	
	r	*p* Value	r	*p* Value	r	*p* Value
GRP	1.00		–0.677	<0.0001	–0.399	<0.0001
VCS	–0.677	<0.0001	1.00		0.647	<0.0001
PP	–0.399	<0.0001	0.647	<0.0001	1.00	

Controlling variables: Age and gender. Coefficient (r); two-tailed test of significance is used. GRP, Gla-rich protein; VCS, vascular calcification score; PP, pulse pressure.

**Table 4:** Factors associated with the vascular calcification score (VCS) and pulse pressure (PP).

Independent Variable	VCS			PP		
	β	OR (95% CI)	*p* Value	β	OR (95% CI)	*p* Value
Age	0.008	1.008 (0.962–1.057)	0.731	–0.001	0.999 (0.948–1.052)	0.969
eGFR	–0.085	0.919 (0.885–0.954)	<0.0001	–0.086	0.917 (0.877–0.959)	<0.0001
P	1.885	6.585 (2.468–17.570)	<0.0001	1.486	4.420 (1.822–10.725)	0.001
Ca	0.508	1.663 (0.796–3.471)	0.176	0.617	1.853 (0.795–4.322)	0.153
CaxP	0.104	1.110 (1.019–1.209)	0.016	0.096	1.101 (1.006–1.205)	0.037
PTH	0	1 (0.996–1.003)	0.778	0.001	1.001 (0.997–1.004)	0.696
FGF-23	0.014	1.014 (1.007–1.022)	<0.0001	0.015	1.015 (1.008–1.022)	<0.0001
α-Klotho	–0.007	0.093 (0.080–0.297)	<0.0001	–0.067	0.784 (0.403–0.889)	0.035
IL-6	0.088	1.192 (1.062–1.238)	0.042	0.1214	1.132 (1.093–1.291)	0.043
GRP	–5.203	0.550 (0.167–0.768)	<0.0001	–5.232	0.105 (0.095–0.378)	<0.0001

Univariate logistic regression analysis. OR, odds ratio; CI, confidence interval; eGFR, estimated glomerular filtration rate; P, phosphate; Ca, calcium; CaxP, calcium (x) phosphate; PTH, parathyroid hormone; FGF-23, fibroblast growth factor 23; IL-6, interleukin 6; GRP, Gla-rich protein; VCS, vascular calcification score; PP, pulse pressure.

**Table 5:** GRP is significantly associated with vascular calcification score (VCS) and pulse pressure (PP).

Independent Variable	VCS			PP		
	β	OR (95% CI)	*p* Value	β	OR (95% CI)	*p* Value
eGFR	–0.064	0.938 (0.900–0.978)	0.003	–0.061	0.941 (0.894–0.990)	0.018
FGF-23	0.011	1.011 (1.003–1.019)	0.006	0.014	1.014 (1.006–1.023)	0.001
GRP	–0.120	0.128 (0.010–0.771)	0.001	–0.024	0.132 (0.098–0.836)	0.004

Multivariate logistic regression, forward stepwise (likelihood ratio) adjusted for age, gender (1), IL-6, P, CaxP, and α-Klotho. OR, odds ratio; CI, confidence interval; eGFR, estimated glomerular filtration rate; FGF-23, fibroblast growth factor 23; GRP, Gla-rich protein; VCS, vascular calcification score; PP, pulse pressure.
